# DOM: Dual Optical Mapping Integrating Sequence‐Specific Barcodes and A/T Density Profiles

**DOI:** 10.1002/smtd.70883

**Published:** 2026-07-22

**Authors:** Jaeyoung Bae, Yunchul Kim, Joohee Choe, Jinhui Hong, Byoungsang Lee, Kaori Hashiya, Toshikazu Bando, Hiroshi Sugiyama, Seonghyun Lee, Jung Heon Lee, Kyubong Jo

**Affiliations:** ^1^ Department of Chemistry Sogang University Seoul South Korea; ^2^ School of Advanced Materials Science and Engineering Sungkyunkwan University Suwon South Korea; ^3^ Institute For Integrated Cell‐Material Science (iCeMS) Kyoto University Kyoto Japan; ^4^ Department of MetaBioHealth Sungkyunkwan University Suwon South Korea; ^5^ Department of Precision Medicine Sungkyunkwan University Suwon South Korea; ^6^ Department of Chemical and Biomolecular Engineering Sogang University Seoul South Korea; ^7^ Center for Nano Materials Sogang University Seoul South Korea

**Keywords:** AT‐specific fluorescent polyamide, cross‐correlation alignment, dual‐channel analysis, optical genome mapping, single‐molecule imaging

## Abstract

Accurate single‐molecule optical genome mapping remains challenging due to the limited information content of barcode‐only or profile‐only strategies. Here, we present Dual Optical Mapping (DOM), which integrates sequence‐specific barcode markers with AT frequency‐dependent intensity profiles on the same DNA molecules to enhance mapping accuracy. Conventional optical mapping approaches rely either on sequence‐specific barcodes generated by restriction endonucleases, nicking enzymes, or methyltransferases, or on dense profile mapping using DNA‐binding molecules. By combining these complementary sources of information within a unified dual‐channel framework, DOM increases positional specificity and alignment confidence. As a model system, we applied DOM to the *E. coli* genome (4.6 Mbp), where the combined dual‐channel scoring metric (cc_rg2) ranked 172 of 182 molecules (95%) at the correct genomic locus as the top match, while 179 of 182 molecules (98%) could be validated after quality‐control review. We further extended DOM to the human genome through genome‐wide cross‐correlation scanning coupled with placement score ranking. This quantitative framework enables objective evaluation of alignment distinctiveness across the entire genome, demonstrating scalability to large and complex genomic contexts. These results establish DOM as a scalable dual‐channel framework for high‐accuracy single‐molecule genome mapping across both bacterial and human genomes.

## Graphical abstract

Dual Optical Mapping (DOM) combines sequence‐specific barcodes and an A/T profile to generate complementary genomic signatures along single DNA molecules. The integration of sparse and dense mapping information enables accurate and confident genome mapping, facilitating reliable localization of long DNA molecules in complex genomes, including the diploid human genome.

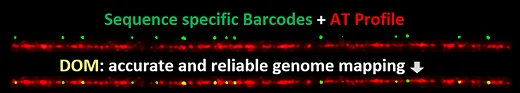

## 1 Introduction

The visualization of DNA sequences has been a long‐standing challenge in biochemistry and genomics. Since the first observation of DNA using electron microscopy in 1948 [1], numerous attempts have been made to visualize the sequences on the DNA molecule, but direct observation of DNA sequences remains a challenge. In 1966, Inman developed DNA denaturation mapping to visualize high AT frequency regions under an electron microscope [2]. Since the development of fluorescent dyes in the 1970s, DNA visualization has moved primarily to fluorescence microscopy [3]. Fluorescence in situ hybridization (FISH) has been used to visualize the position of specific sequences in the metaphase chromosome [4] and was further developed to fiber‐FISH [5].

In 1993, Schwartz and colleagues invented the single‐molecule DNA optical mapping system, in which elongated DNA molecules were sequence‐specifically digested by restriction enzymes [6]. In the original restriction‐based optical mapping workflow, long DNA molecules were uniformly stained with intercalating dyes such as YOYO‐1 and digested by restriction endonucleases while elongated on a surface. The resulting fragment pattern was visualized by fluorescence microscopy, and the distances between cut sites were used to construct genome‐wide restriction maps. This platform has been used for de novo sequence assembly and structural rearrangement analysis of the human genome [7]. Beginning in 2007, optical mapping systems advanced to the next platform, in which DNA was sequence‐specifically labeled by nicking enzymes and polymerase with fluorochrome‐conjugated nucleotides [8, 9, 10]. Alternatively, sequence‐specific methyltransferase‐directed DNA labeling approaches have been developed, including the mTAG strategy, in which methyltransferases transfer functional groups from synthetic S‐adenosyl‐L‐methionine (SAM) analogues to specific recognition sites [11]. These modified bases can subsequently be conjugated to fluorophores for optical genome mapping [12, 13]. These sequence‐specific labeling methods enabled analysis of DNA molecules confined in nanochannels, forming the basis of the commercialized Bionano Genomics platform [14]. Currently, it is one of the essential long‐read DNA analysis tools that enabled the first complete gapless telomere‐to‐telomere assembly of the human haploid genome [15], and the human diploid genome [16]. A key feature of optical mapping systems is their capacity to provide sequence‐dependent information from long DNA molecules. However, because barcode‐based labeling provides discrete site‐specific signals rather than continuous nucleotide‐level readout, the amount of recoverable sequence information remains fundamentally limited compared with sequencing platforms. As a result, a substantial fraction of imaged DNA molecules cannot be confidently aligned to the reference genome or a de novo assembled map, thereby reducing the overall alignment rate.

One approach to enrich more detailed information from observed DNA images is to use densely stained DNA intensity profiles. Unlike barcode‐type mapping, these profiles provide more detailed and contextual sequence information, as each base pair contributes to the overall intensity pattern. In 2010, Reisner et al. reported DNA denaturation mapping that utilizes the partial melting of YOYO‐1‐stained DNA [17]. An increase in temperature and formamide concentration preferentially denatures DNA strands at AT‐rich regions, releasing YOYO‐1 from DNA, similar to Inman's denaturation mapping under an electron microscope [2]. Alternatively, Nyberg et al. reported another approach using netropsin, which specifically binds to AT base pairs and blocks the intercalation of YOYO‐1 into DNA; thus, the fluorescence intensity profile of stained DNA indicates the ratio of GC base pairs [18, 19]. As an extension of this method, we reported in 2018 an approach that used a fluorophore‐linked pyrrole octamer as an AT‐specific DNA staining agent [20]. Like netropsin, the pyrroles in polyamides form hydrogen bonds with the minor groove of AT base pairs. This profile mapping approach is advantageous because it provides spatially continuous sequence‐composition information at optical resolution, within the limits imposed by the point spread function. However, the major challenge of this approach was aligning DNA images stained in an AT‐frequency–dependent manner to a large genome map, since many regions of the genome exhibited similar patterns with AT‐dependent staining alone. When the highest and second‐highest cross‐correlation values were not clearly distinct, confident determination of the correct alignment became difficult.

One possible strategy to overcome this limitation is to combine dense sequence‐composition information with sparse sequence‐specific labels. Previous studies have demonstrated that integrating dense and sparse optical DNA mapping modalities can improve alignment confidence [21]. In this study, we developed Dual Optical Mapping (DOM), which integrates DLE‐1 sequence‐specific labeling and AP8‐mediated AT‐frequency profiling [22, 23]. By integrating these complementary strategies on the same DNA molecules, DOM improves the reliability and interpretability of single‐molecule DNA image analysis across whole genomes. Unlike conventional barcode‐based methods that may leave a subset of molecules ambiguously ranked, DOM incorporates AT‐specific intensity profiles to enhance locus discrimination. We applied DOM to two model systems: for the *E. coli* genome, the cc_rg2 metric (cc_rg2 = cc_r × cc_g^2^) correctly identified 172 of 182 molecules (95%) as the top‐ranked matches, while 179 of 182 molecules (98%) could be validated after review of candidate alignments. We further extended DOM to the human genome using genome‐wide cross‐correlation scanning and placement score‐based ranking, demonstrating scalability of the dual‐channel framework to large and complex genomes.

## 2 Results and Discussion

### 2.1 AT‐Rich DNA Recognition by ATTO647N–Pyrrole Octamer (AP8)

**FIGURE 1:**
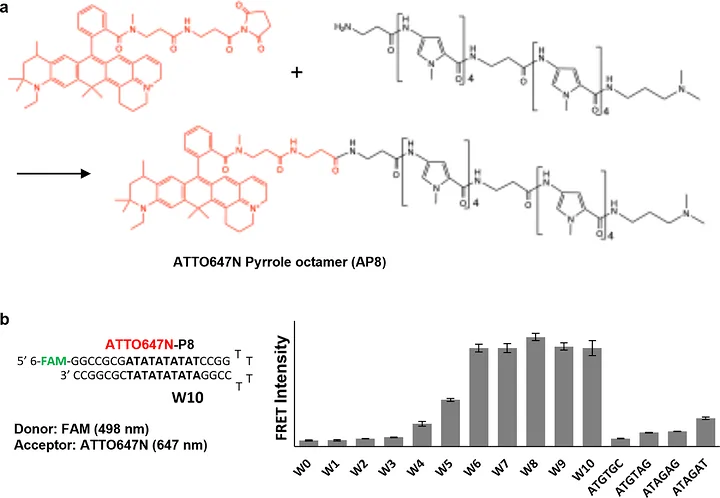
ATTO647N‐P8 (AP8) for AT‐specific staining. (a) Synthesis of ATTO647N‐conjugated pyrrole octamer (AP8). The ATTO647N fluorophore was covalently coupled to the N‐terminal amine of a pyrrole octamer (P8) through an amide‐forming condensation reaction, yielding AP8, an AT‐specific DNA staining reagent. (b) Fluorescence resonance energy transfer (FRET) assay to evaluate the binding affinity of AP8. (Left) W10 DNA probe used in the FRET assay; W denotes a weak base (A or T), and the putative AP8 binding sites within the AT‐rich region are indicated in bold. (Right) FRET intensities measured for various putative binding sequences. Error bars represent the standard deviation from *N* = 3 independent experiments.

ATTO647N–pyrrole octamer (AP8) preferentially binds to AT‐rich regions, as demonstrated in our previous studies [20, 22, 24, 25]. Pyrrole–imidazole polyamides (PIPs) are well known to exhibit sequence‐selective DNA recognition, with pyrrole‐rich motifs preferentially binding to weak bases (represented as W, denoting A or T) in the minor groove of the DNA double helix [26, 27]. The pyrrole octamer (P8) was synthesized via computer‐assisted Fmoc solid‐phase synthesis [28], followed by conjugation with an ATTO647N NHS ester to render the molecule fluorescent (Figure 1a). We chose ATTO647N as a fluorophore since it is one of the brightest commercially available organic dyes (*ϵϕ* = 97.5 mM^−1 ^cm^−1^) [29, 30].

Figure 1b presents the systematic analysis of the DNA‐binding characteristics of AP8. For this purpose, we conducted a fluorescence resonance energy transfer (FRET) experiment [31, 32] using AP8‐bound DNA oligonucleotides labeled with fluorescein amidite (FAM) at the 5′ end, varying the number of W (A or T) bases. Upon excitation at 498 nm, FAM (acting as the donor) transfers its resonance energy to ATTO647N (functioning as the acceptor). The bar graph reveals that the FRET efficiency starts increasing from W4 and reaches a plateau at W6 (5′‐ATATAT‐3′). Compared to the W6 FRET intensity, W4 and W5 exhibit reduced intensities of 18.1% and 43.9%, respectively. These results align with earlier studies: dimeric pyrrole‐imidazole trimers select five base pairs [33] and dimeric pyrrole‐imidazole tetramers selectively bind to six base pairs [34]. Thus, AP8 is expected to bind approximately six base pairs, consistent with the established binding behavior of dimeric pyrrole polyamides. Subsequently, we assessed the influence of guanine (G) within the hexamer binding sites and observed a reduction in binding affinity. Compared to W6, the FRET intensity for ATAGAT was reduced to 24.1%. The introduction of an additional G in the hexamer, as seen in sequences like ATGTAG and ATAGAG, further decreased the FRET intensities to 8.4% and 9.8%, respectively. Notably, the sequence ATGTGC exhibited only minimal binding affinity, with the FRET intensity reduced to 2.0% relative to W6. Taken together, these results suggest that W6 predominantly functions as the binding site for AP8, while displaying a lesser binding affinity for other sequences containing W. Importantly, AP8 binding does not represent a simple binary (on/off) interaction; rather, it reflects a sequence‐dependent continuum of binding affinities, indicating that its recognition behavior is inherently complex and not reducible to digital information.

### 2.2 AT Frequency Reference Map From the Genome Sequence

**FIGURE 2:**
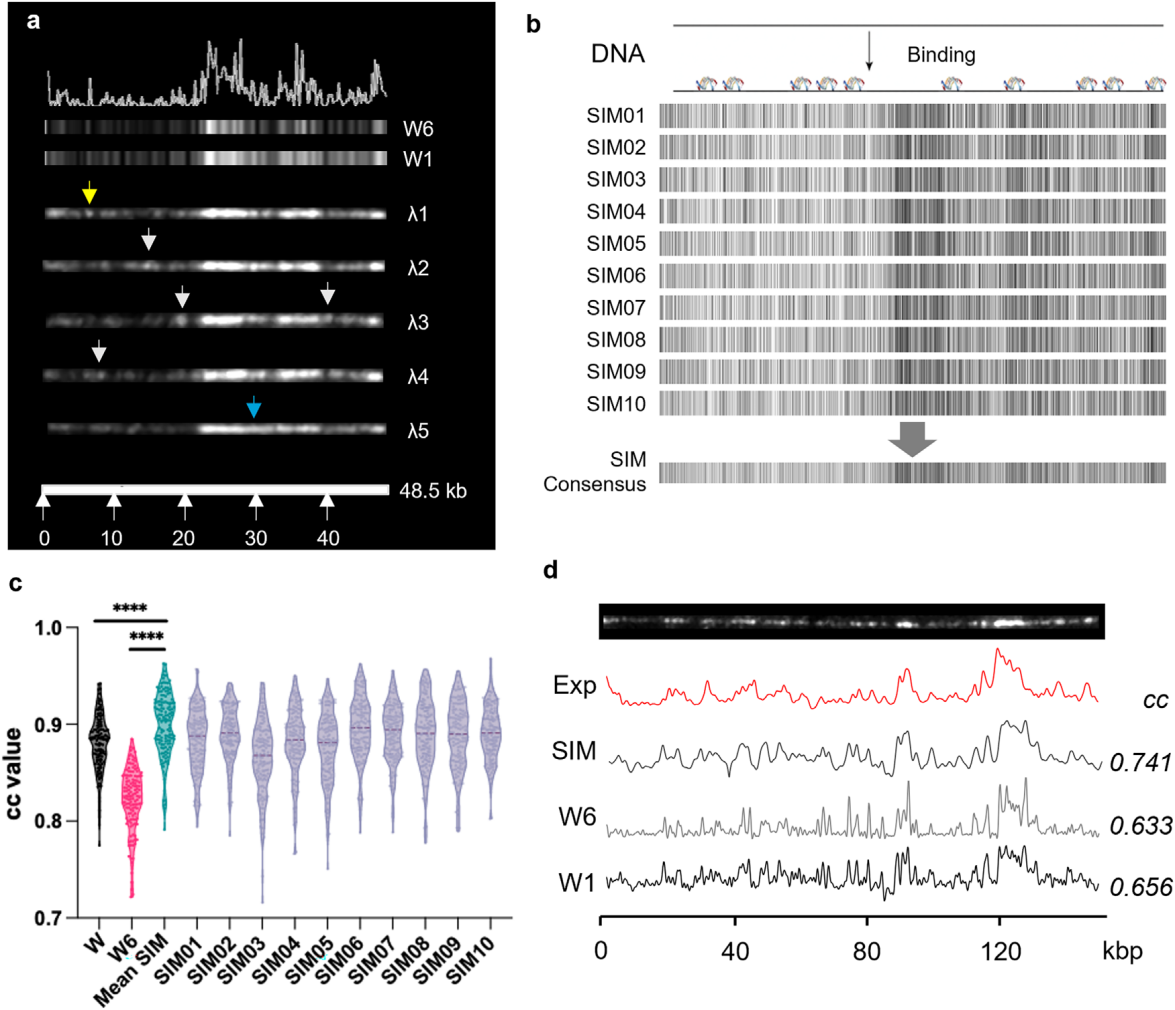
Quantitative characterization of AP8 binding to DNA. (a) Frequency profiles of six consecutive weak bases (W = A or T; W6) and W1 across the λ DNA genome, shown as image‐like maps based on 200 bp divisions. Corresponding single‐molecule DNA images are shown below. Arrows indicate characteristic features observed in individual molecules. (b) Schematic representation of the AP8 binding simulation. Ten independent simulation realizations (SIM01‐SIM10) generated from the λ DNA sequence are shown. The consensus map was obtained by averaging the simulated binding profiles. (c) Violin plots showing Pearson cross‐correlation coefficients (cc values) between experimental images (*N* = 133) and the SIM maps from (b). ****p < 0.0001 by Student's *t*‐test. (d) Comparison of the consensus SIM map, W6, W1, and experimental AP8 fluorescence intensity obtained from a DNA fragment derived from *E. coli* MG1655, with the corresponding cc values indicated on the right. Additional supporting data are provided in Figure S1.

Based on the binding characteristics investigated in Figure 1b, we calculated the frequency of W6 motifs in the sequence by dividing it into 200 bp segments, corresponding to the pixel resolution of our microscopic system. Figure 2a presents a white‐color profile representing the W6 frequency per 200 bp in the bacteriophage λ genome (48.5 kb), together with image‐like DNA maps representing W6 and W1 frequency, as well as fluorescence microscope images of λ DNA molecules stained with AP8. Five λ DNA molecules (λ1–λ5) were fully elongated to achieve near full‐length extension through tethering, elongating, and drying, following the previous reports [25, 35]. Briefly, DNA molecules were tethered at one end to a glass surface and stretched by capillary flow. Subsequent air‐assisted drying immobilized the elongated molecules on the surface, yielding near full‐length extension close to the theoretical contour length of B‐form DNA. Despite their similar overall contour lengths and degrees of stretching, variations in fluorescence intensity profiles were observed among the captured DNA images. For example, λ1 DNA shows a dim spot at the 6.3 kb region (yellow arrow), which is absent in λ2. In addition, λ5 exhibits a smoother fluorescence profile around the 30 kb region (cyan arrow) compared with other molecules. Roughly, the five λ DNA images are consistent with the W6 and W1 maps, but they differ in local details. These observations indicate that stochastic binding behavior remains an intrinsic feature of single‐molecule experiments, posing a potential limitation for accurate analysis. Therefore, while AT‐profiles provide valuable sequence‐composition information, they cannot by themselves resolve genomic position with high confidence, especially when molecule‐to‐molecule binding variability yields non‐deterministic intensity profiles.

Figure 2b presents computer simulations of AP8 binding designed to visualize stochastic binding realizations and the resulting consensus profile, rather than to reproduce the optical appearance of experimental fluorescence images. We hypothesized that when AP8 binds to DNA, it could hinder adjacent binding events due to steric hindrance. Accordingly, a bound AP8 molecule was assumed to occlude approximately 10 base pairs, preventing neighboring binding events within this region. In addition, the simulations incorporated AP8 binding to sequences containing G or C according to the experimentally measured binding affinities shown in Figure 1b. From these simulations, we generated image‐like DNA maps (SIM01–SIM10). While the individual simulated images were broadly similar, each displayed subtle variations. Averaging all ten simulations resulted in a consensus reference map (SIM Consensus in Figure 2b). The Python code for sim2RGmap.py is provided in the GitHub repository (https://github.com/jolaboffice/DOM/).

We next investigated whether the simulation‐based consensus map was more accurate than the sequence frequency‐based in silico map. To this end, we computed Pearson cross‐correlation (cc) values between the experimental intensity profile and the in silico AT profile maps. The cc values of 133 λ DNA molecule images were calculated against W1, W6, the SIM consensus map, and the individual simulations (SIM01–SIM10). The average cc value obtained using λ DNA image clips and W1 map was 0.880 ± 0.00258, whereas that with W6 was 0.822 ± 0.00283. Notably, the cc value with the SIM consensus map further increased to 0.900 ± 0.00288 (Figure 2c). These results indicate that molecular binding simulations can generate a more accurate reference map for AP8 staining intensity, despite variation in individual simulations. In addition to λ DNA, we then applied this approach to an *E. coli* DNA fragment (Figure 2d), where the cc value with the SIM consensus map was 0.741, compared to 0.633 and 0.656 for W6 and W1, respectively. To generalize this result, we calculated cc_r values for all top‐ranked molecules using both the full‐length region and the central 50 kb segment, demonstrating that the SIM reference consistently outperforms W1 and W6, as shown in Figure S1.

Although the SIM‐based reference improves correlation accuracy compared with simple W1 or W6 frequency maps, the alignment still relies exclusively on sequence‐composition information. Even when refined by stochastic binding simulations, AT‐profile–based mapping lacks sufficient positional specificity to uniquely determine genomic coordinates in large and repetitive genomes. Consequently, an orthogonal source of sequence‐specific information is required. This limitation motivated the development of DOM, which integrates discrete barcode markers with AT‐frequency profiles to achieve robust and high‐confidence genome alignment.

### 2.3 Dual Optical Map (DOM)

**FIGURE 3:**
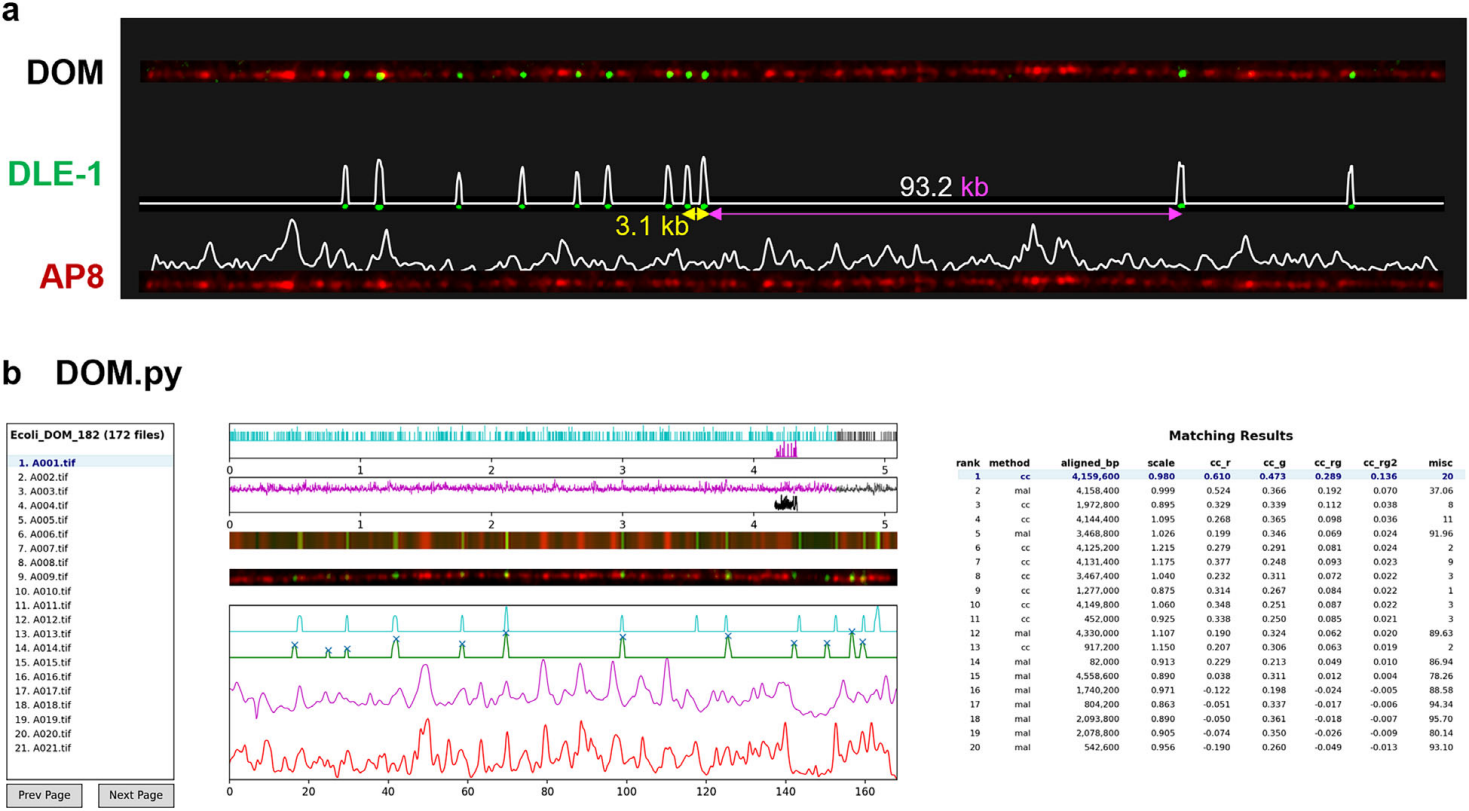
Dual Optical Mapping (DOM) and alignment using DOM.py. (a) Representative DOM image showing green fluorescence from sequence‐specific barcode labels and red fluorescence from AT‐specific staining. (b) DOM.py program interface. For each alignment candidate, the reference and experimental profiles are displayed. The matching table reports the rank, alignment method (cc or Maligner [36]), aligned_bp, scale, cc_r, cc_g, cc_rg (= cc_r × cc_g), cc_rg2 (= cc_r × cc_g^2^), and miscellaneous (misc) information, and is sorted by cc_rg2 in descending order. For the cc method, the misc column indicates the number of merged alignment candidates: during cross‐correlation scanning, when multiple nearby genomic positions yield similar cc values, these positions are consolidated by retaining only the highest‐scoring candidate. For the mal method, the misc column reports the penalty score assigned by the Maligner dynamic‐program (maligner_dp). By clicking a file name in the left panel or a matching result in the right panel, the center panel updates accordingly.

Figure 3a illustrates a representative DOM image of a DNA molecule, in which green puncta mark CTTAAG barcode sites labeled by DLE‐1, and the red signal indicates AT‐rich regions recognized by AP8. Conventional sequence‐specific optical mapping methods rely on sparse barcode labels generated by nicking‐ or methyltransferase‐based labeling strategies [8, 9, 10, 11, 12, 13]. While these approaches provide robust sequence anchors, long unlabeled intervals frequently remain between adjacent labels. For example, the magenta arrow in Figure 3a indicates a 93.2‐kb region without any detectable barcode signal, whereas the yellow arrow corresponds to a 3.1‐kb region. When aligned to the reference genome, this 93.2‐kb interval contains 91 genes (Figure S2). Such long unlabeled regions fundamentally limit the spatial information content available for alignment and genome analysis.

These limitations motivated the development of DOM, which combines the strengths of both labeling approaches. In sparse‐labeling methods, false‐negative or false‐positive events critically affect the accuracy of the optical map because each label carries substantial weight in the alignment. In contrast, dense labeling is inherently more tolerant to such errors; even if a portion of the labels is missing or inaccurately detected, the overall profile remains largely reliable due to the abundance of data points. However, as shown in Figure 2, the AT‐profile labeling exhibits single‐molecule variability originating from random events, which limits reproducibility and makes straightforward interpretation challenging. By combining a simple and robust sparse labeling pattern with a dense AT‐profile signal, DOM leverages the strengths of both approaches and compensates for their individual weaknesses. Consequently, sparse labels provide reliable genomic anchors, while the dense AT‐dependent profiles supply greater spatial information density, enabling more robust and reproducible single‐molecule genome analysis.

### 2.4 Alignment of the DOM Image to the Reference Map

Figure 3b presents a representative DOM.py output showing a DNA molecule aligned to the *E. coli* reference map. The DOM.py source code is available in our GitHub repository (https://github.com/jolaboffice/DOM). The program displays the aligned genomic position along with the in silico reference map (cyan for barcode sites and magenta for the AT‐dependent profile), the experimental data image, and their corresponding intensity profiles. DOM.py computes the combined score cc_rg2 (= cc_r × cc_g^2^) for candidate genomic positions and ranks them accordingly. A previous dual‐modality OGM framework transformed modality‐specific alignment scores into *p*‐values and combined them using statistical significance measures such as Stouffer's method [21]. In contrast, DOM directly combines channel‐specific correlation metrics (cc_r and cc_g) into a multiplicative ranking score (cc_rg2) for prioritizing candidate genomic alignments.

For this calculation, we used Pearson cross‐correlation (PCC) to the continuous AT‐intensity profile and differential cross‐correlation (DCC) [21] to the discrete sequence‐specific labels, the latter being well suited to capturing the positional nature of the green‐label signal. In this framework, cc_r and cc_g quantify alignment agreement for the red and green channels, respectively. Each coefficient measures the correspondence between the experimental signal and its reference counterpart. Because the two channels are derived from distinct labeling modalities and imaging conditions, their noise characteristics are expected to be largely independent. The product cc_rg = cc_r × cc_g serves as a heuristic combined score that favors alignments in which both channels exhibit high correlation values. This multiplicative combination penalizes candidate alignments when either cc_r or cc_g is low. In this implementation, equal weighting of the two channels was used without introducing additional fitted parameters. However, our data analysis indicated that cc_g contributes more strongly to the alignment than cc_r. This difference likely stems from the fact that cc_g reflects a sequence‐specific biochemical reaction forming covalent bonds, whereas cc_r originates from non‐covalent AT‐affinity binding. Accordingly, we introduced a weighted metric, cc_rg2 = cc_r × cc_g^2^, which preferentially amplifies the contribution of the green‐channel signal. The DOM.py output in Figure 3b includes the full alignment table, listing the rank, method, aligned bp, stretch factor (0.8–1.2), cc_r, cc_g, cc_rg, cc_rg2, and associated metadata denoted as misc. The list is sorted in descending order of cc_rg2, facilitating identification of high‐confidence genomic alignments based on ranking.

In DOM.py, we additionally integrated Maligner, a dynamic programming‐based aligner downloaded from GitHub (https://github.com/LeeMendelowitz/maligner), to perform comparative analysis of barcode‐based alignment results [36]. For each candidate locus proposed by Maligner, we extracted the aligned genomic position and stretching factor, and subsequently computed cc_r, cc_g, cc_rg, and cc_rg2 values using the same alignment parameters. Candidate loci were then re‐ranked according to cc_rg2 to enable direct comparison between barcode‐only alignment and DOM scoring.

In the example shown in Figure 3b, a total of twenty candidate alignments are displayed, comprising ten candidates obtained from Maligner and ten candidate loci ranked by the cc_rg2‐based scoring scheme. The second‐ranked Maligner hit corresponds to an aligned position at 4,158,400 bp, whereas the cc‐based approach identified 4,159,600 bp as the first, resulting in a positional difference of approximately 1.2 kb. The close agreement between the top‐ranked locus identified by cc_rg2 and the best match reported by Maligner indicates that both approaches converge on a highly similar genomic location despite using different scoring strategies. The dynamic programming–derived penalty score calculated by Maligner is indicated in the “misc” column; among entries labeled with method = mal, the lowest penalty score (37.06) corresponds to the best matching position according to Maligner.

### 2.5 Genome‐Wide Implementation of DOM

**FIGURE 4:**
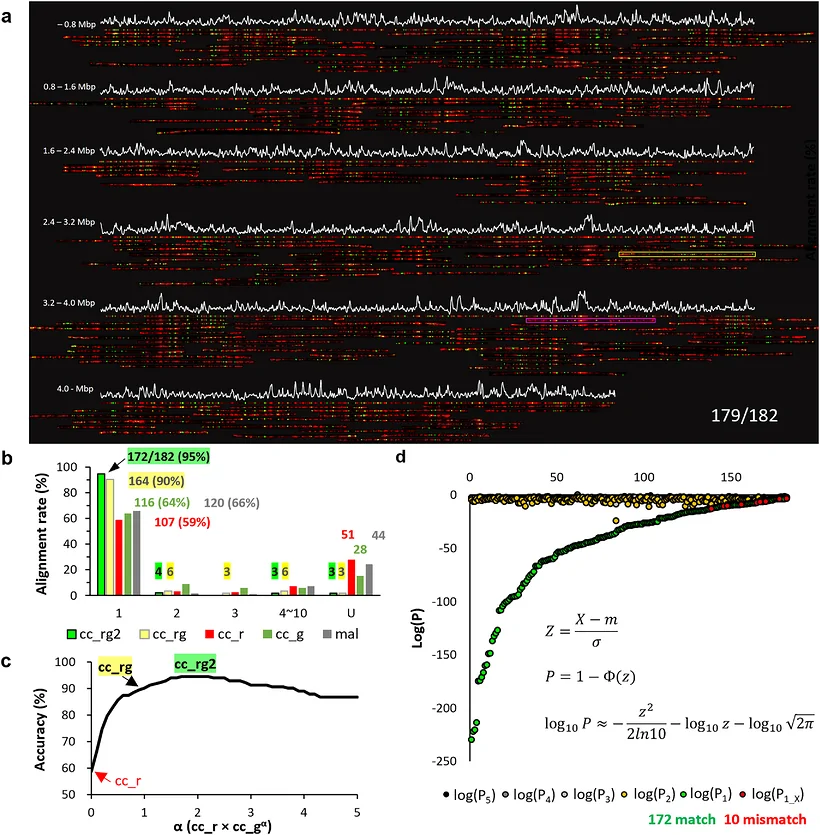
Genome‐wide DOM on *E. coli* K‐12 MG1655. (a) Alignment of 179 DNA images out of 182 experimentally acquired images. The white trace represents the in silico SIM map of the *E. coli* genome. Colored boxes indicate DNA molecules analyzed in Figure 5a (magenta) and 5b (yellow). (b) Comparison of alignment performance across five metrics, plotted in the following order: cc_rg2 (light green), cc_rg (yellow), cc_r (red), cc_g (dark green), and Maligner (gray). The numbers in the graph indicate the number of DNA molecules for each case. The x‐axis shows the rank, and “U” denotes undetermined. (c) Optimization of the weighting exponent α in the combined score cc_r × cc_gα, where α = 0, 1, and 2 yield cc_r, cc_rg, and cc_rg2, respectively. (d) Genome‐wide placement score analysis of DOM‐based alignments in *E. coli* genome. Distribution of log‐transformed placement scores (log(P), where P denotes the genome‐wide placement score) across all genomic positions and stretching factors (0.8–1.2) calculated for each of the 182 DNA molecules. For most molecules, the log(P_1_) value (corresponding to the highest cc_rg2 peak) is clearly separated from log(P_2_) and from all lower‐ranked log(P) values, demonstrating strong separation between the top‐ranked cc_rg2 candidate and lower‐ranked alternatives. The ten mismatched cases are highlighted in red (log(P_1_X_)).

As a model system, we applied DOM to the 4.6‐Mbp genome of *E. coli* MG1655. To minimize reference ambiguity, we first generated a strain‐specific reference assembly from the same laboratory *E. coli* culture used for DOM experiments using Illumina/ONT sequencing, rather than relying solely on a public reference genome. This assembly was then used to generate the in silico DOM reference map. Figure 4a summarizes the results, showing 179 DNA molecules successfully aligned to the reference genome. A total of 182 molecular images longer than 100 kb were initially obtained, and DOM.py generated alignment results for all molecules. The analyzed molecules were typically long relative to the genome size (N50 = 153 kb, corresponding to approximately 3.3% of the 4.6‐Mb genome), reducing the likelihood of multiple equally plausible genomic placements. Each of the twenty candidate alignments proposed by the DOM.py interface was manually reviewed to confirm consistency with the strain‐specific reference assembly (Figure 3b). Three molecules were excluded after repeated alignment attempts, including manual segmentation of molecular images, resulting in 179 validated alignments (98% of the initial dataset). These results demonstrate that DOM enables accurate identification of the vast majority of properly extended DNA molecules. Although manual inspection ensured rigorous validation in this study, the placement score framework introduced here provides a basis for future automated filtering and quality‐control in high‐throughput applications. In future high‐throughput implementations, molecules with insufficient separation between the top‐ranked and alternative candidates could be automatically excluded using placement score–based criteria.

Figure 4b compares the performance of various alignment methods (cc_rg2, cc_rg, cc_r, cc_g, and Maligner) against manually validated alignments to the strain‐specific *E. coli* reference assembly. DOM using cc_rg2 produced 172 first‐matched images (95%), whereas using cc_rg yielded 164 (90%). In contrast, single‐channel methods based solely on dense or sparse optical mapping resulted in 107 (59%), 116 (64%), and 120 (66%) first matches. This improvement is consistent with simulation results that Torstensson et al. previously reported [21]. Although the first‐match results from cc_g (116) and Maligner (120) appear similar—reflecting their reliance on the same underlying barcode data—they differ conceptually: cc_g employs DCC [21], whereas Maligner uses a dynamic‐programming algorithm based on penalty scoring [36]. The remaining molecules were aligned to the second–10th ranked positions, whereas the rest were categorized as undetermined. The markedly lower number of undetermined molecules for DOM (3) compared to single‐channel methods (51, 28, 44) further underscores its robustness.

As shown in Figure 4b, the DOM analysis using cc_rg2 achieves a higher alignment rate compared to the cc_rg formulation. This observation motivated us to examine whether the exponent on cc_g is optimally set at 2. We therefore varied the exponent α from 0 upward in increments of 0.1 to 5 and evaluated performance across conditions (Figure 4c). Here, α = 0 corresponds to using cc_r alone, and α = 1 corresponds to the cc_rg metric. The results demonstrate that α = 2 provides the highest accuracy, supporting the use of a squared weighting of the cc_g term.

We also considered defining an absolute threshold applicable to all data images. However, such a threshold is not feasible because the absolute values of cc_r and cc_g vary substantially between molecules due to differences in stretching, local sequence composition, and noise. Consequently, direct comparison of absolute correlation values across molecules seems unreliable. For this reason, DOM.py adopts a threshold‐free, ranking‐based approach in which the genomic position with the highest combined score (cc_rg2) is selected as the alignment result. This ranking strategy reduces sensitivity to molecule‐to‐molecule variability and avoids the use of arbitrary cutoff parameters; in practice, it performed robustly across our bacterial dataset.

Instead, as shown in Figure 4d, we calculated a genome‐wide placement score (P) for each candidate alignment. For every molecule, we estimated the genome‐wide background distribution of cc_rg2 values by scanning genomic positions across the 4.6‐Mbp genome and calculating the mean and standard deviation of the resulting cc_rg2 scores. These parameters were then used to calculate the corresponding placement score for each candidate alignment (Figure S4). Strictly speaking, the distribution of cc_rg2 does not perfectly follow a normal distribution. While the cc_r component approximates Gaussian behavior, the cc_g term (based on DCC) exhibits a right‐skewed tail. Consequently, the combined cc_rg2 metric shows mild right‐tailed behavior rather than strict symmetry. Therefore, the reported placement scores should be interpreted as measures of alignment distinctiveness derived from genome‐wide null sampling, rather than as formal parametric probabilities under ideal Gaussian assumptions.

In Figure 4d, the top‐ranked cc_rg2 match is indicated by a green circle, and the y‐axis represents the log(P). We also computed log(P) for the second‐ through fifth‐ranked candidates for comparison. The ten cases in which cc_rg2 did not yield the correct top‐ranked match are highlighted in red. For most molecules in this dataset, the placement score associated with the top‐ranked candidate was well separated from those of lower‐ranked candidates. Approximately 20 molecules showed partial overlap among the top five candidates, and in a few of these cases the top‐ranked match did not correspond to the correct genomic position. In one instance, a second‐ranked alignment exhibited a similarly low placement score, which may occur when two genomic regions share similar sequence patterns. This analysis illustrates the ranking behavior of cc_rg2 under the present experimental conditions, rather than providing a formal statistical guarantee of alignment correctness.

**FIGURE 5:**
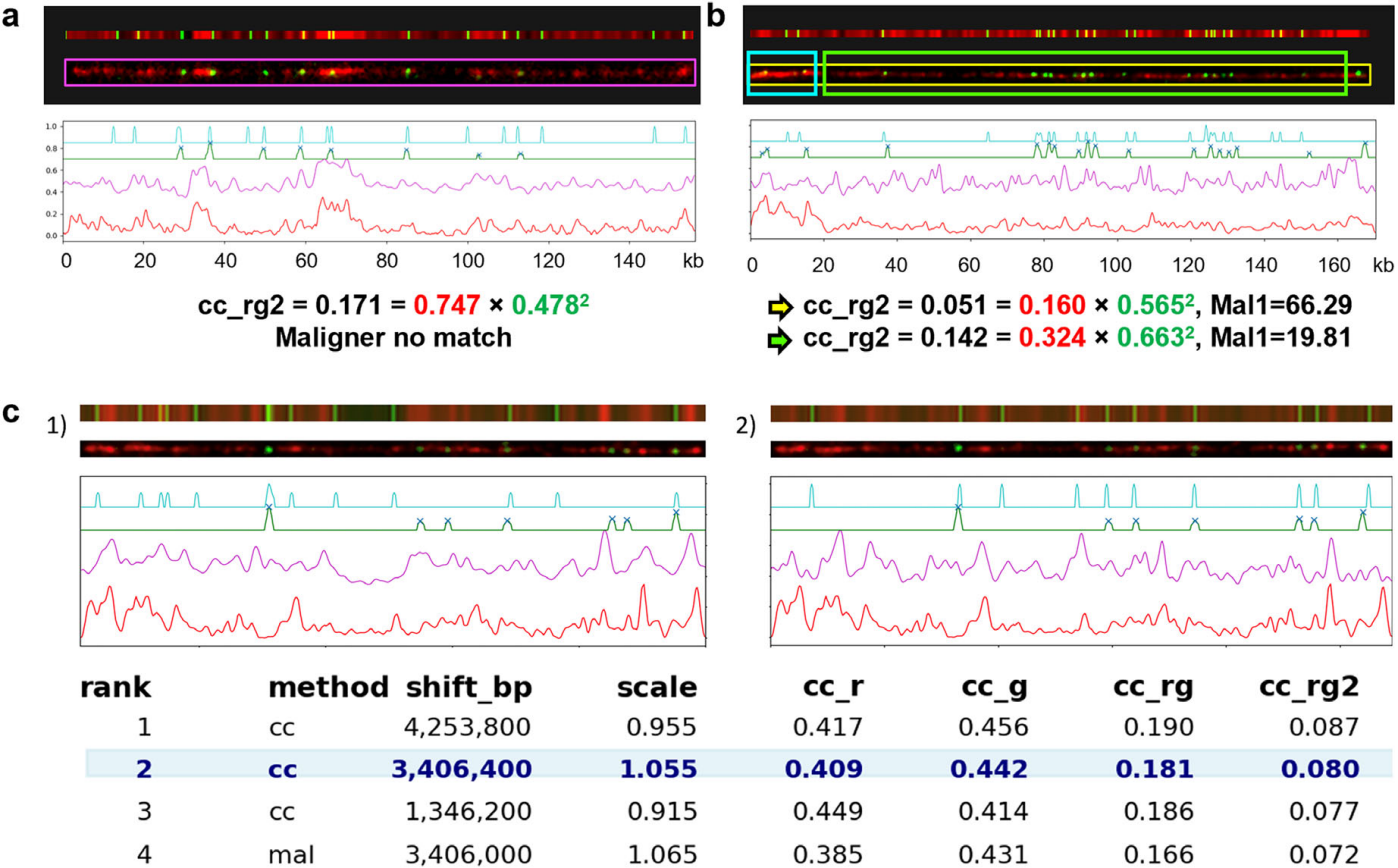
Complementary strengths of barcode labeling and AT‐profile staining in DOM alignment. (a) An example in which insufficient barcode labeling causes Maligner to misalign the molecule, whereas cc_rg2 correctly identifies the genomic position. (b) Example in which local folding or partial stretching distorts the AT‐intensity profile, resulting in low cc_rg2 values; however, the barcode labels remain reliable, and Maligner identifies the correct alignment. The green cropped box shows the top‐ranked match, indicated by the green arrow. (c) Example of a molecule for which the top two cc_rg2 candidates differ only slightly; combined inspection of the AT‐profile, barcode labels, and Maligner ranking resolves the correct genomic position.

Figure 5 provides detailed examples of several notable cases. In Figure 5a, the molecule contains only 8 of the 16 expected barcode labels, causing Maligner to misidentify the genomic position. Nevertheless, the red‐channel AT‐profile closely matches the AT‐dependent reference, and cc_rg2 ranks the correct genomic location as the top candidate, demonstrating that the dense AT‐profile can rescue alignments when the barcode pattern is sparse or partially missing. In contrast, Figure 5b shows a case in which local folding or incomplete stretching distorts the AT‐intensity profile, reducing the red‐channel correlation and lowering the cc_rg2 rank. However, the barcode pattern remains sufficiently preserved outside the folded region, allowing Maligner to identify the correct genomic position, thereby demonstrating that barcode‐based alignment compensates when the AT‐profile is compromised by structural artifacts. Importantly, when the folded ∼20‐kb region at the left end of the molecule is cropped, and the alignment is re‐run, cc_rg2 also promotes the correct position to rank 1, confirming that the discrepancy arose from image distortion rather than from a limitation of the cc_rg2 metric. Additional examples of distortion‐induced misalignment and its correction by cropping are shown in Figure S3. Figure 5c illustrates a molecule for which the top two cc_rg2 candidates differ only slightly, rendering AT‐profile‐based ranking alone insufficient. In this case, the Maligner output at rank 4 independently supports the same genomic position, and the combined evidence from cc_rg2 and Maligner increases confidence in the alignment. Collectively, these examples demonstrate that DOM leverages the complementary strengths of cc_rg2 and Maligner, yielding more consistent alignment results than either modality alone under the present conditions.

### 2.6 DOM Applied to the Human Genome

**FIGURE 6:**
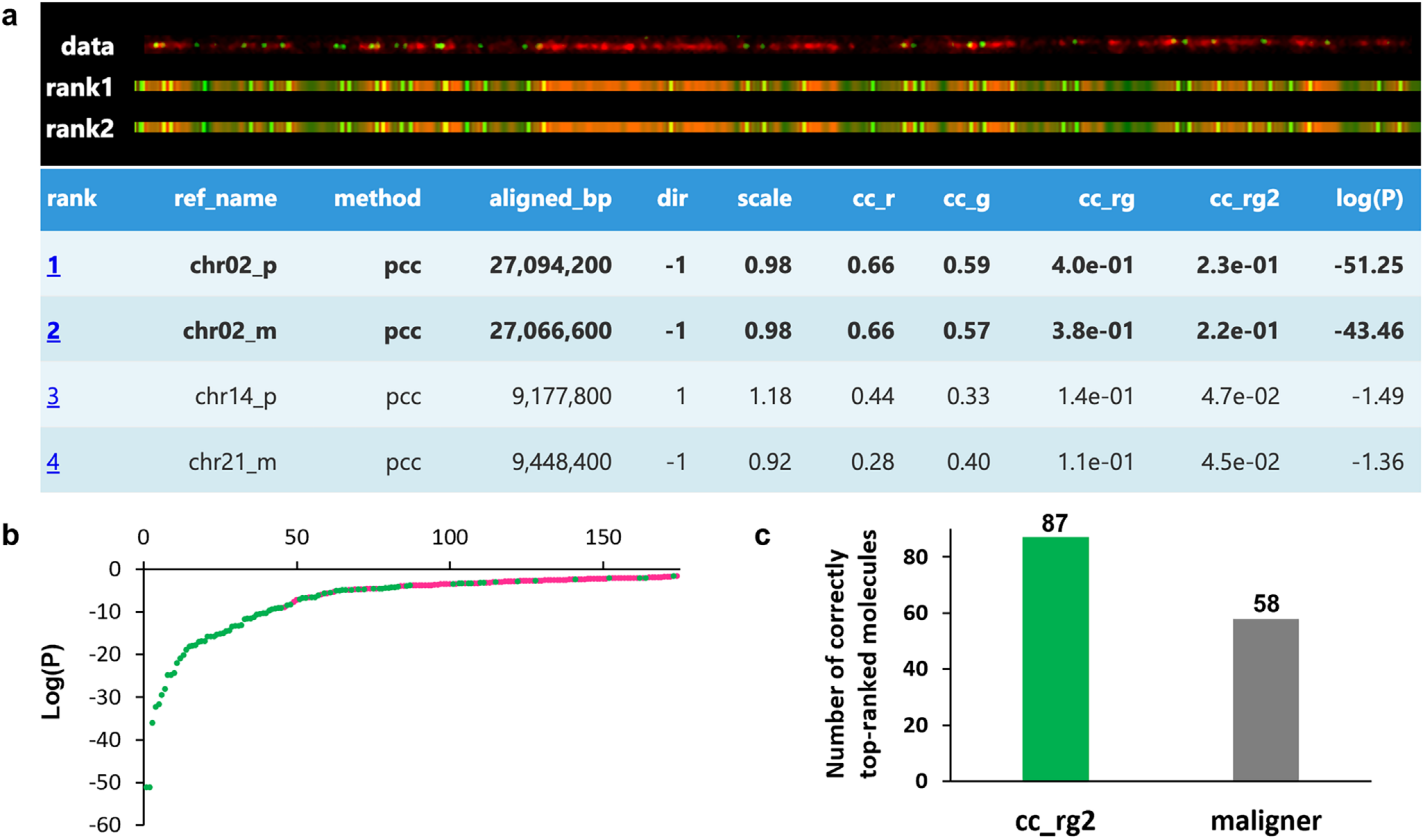
Genome‐wide alignment performance of hDOM. (a) Representative hDOM analysis report for a 252.4 kb DNA molecule. The top panel shows the experimental dual‐color barcode (data) and the top two ranked reference matches (rank 1 and rank 2). The table summarizes genome‐wide alignment results across all 46 chromosomes, including reference name (ref_name), alignment position (aligned_bp), orientation (dir), scale factor (scale), correlation coefficients (cc_r, cc_g), combined scores (cc_rg, cc_rg2), and genome‐wide placement scores expressed as log(P). Candidate loci were ranked by log(P), and the locus with the lowest log(P) value was selected as the final alignment. (b) Genome‐wide distribution of log(P) values for candidate loci. Green dots indicate true alignments confirmed by manual inspection, whereas pink dots represent false matches. (c) Comparison of correctly top‐ranked DNA molecules identified by cc_rg2 (87/174=50%) and maligner (58/174=33%).

We next applied DOM to human genomic DNA from the HG02982 cell line (Figure 6). Because a complete reference genome for HG02982 is not available, we used the diploid human assembly hg002v1.1 (maternal and paternal haplotypes; hg002v1.1.mat and hg002v1.1.pat) as the recently completed reference [37]. To extend DOM analysis to the human genome, we implemented an adapted computational framework termed hDOM (human Dual Optical Mapping), in which cross‐correlation calculations were accelerated on a CUDA‐based GPU computing and Fast Fourier Transform (FFT)‐based convolution to achieve scalable genome‐wide analysis (https://github.com/jolaboffice/DOM/tree/main/hDOM). The Python program hDOM_calc.py computed cc_rg2 values for each imaged DNA molecule against all 46 chromosomes of the reference assembly. Candidate loci were evaluated using a genome‐wide placement score derived from the cc_rg2 distribution for each molecule. The mean and standard deviation of the cc_rg2 values across all 46 reference chromosomes were used to compute molecule‐specific placement scores. Candidate genomic positions were ranked accordingly, and the locus with the lowest placement score was selected as the final alignment.

Figure 6a shows a representative hDOM analysis report for a single 252.4 kb molecule. The experimental green barcode and red intensity profile shown in the “data” panel exhibit strong overall concordance with both rank 1 and rank 2 reference alignments, indicating robust positional agreement across the entire molecule length. Because the human genome is diploid and composed of homologous chromosome pairs with highly similar sequences, hDOM typically identifies two top‐scoring loci corresponding to the paternal and maternal homologs. The presence of these paired high‐confidence matches provides internal consistency and strengthens alignment confidence. In this example, the molecule was mapped to chromosome 2, with slightly different coordinates for the paternal (chr02_p) and maternal (chr02_m) assemblies. These two top‐ranked candidates are clearly separated from rank 3 and rank 4 in both the combined score (cc_rg2) and placement score (log(P)), demonstrating strong discriminative power. Notably, the aligned_bp values for the paternal and maternal chromosomes are not identical. This difference reflects genuine biological variation rather than alignment uncertainty, as homologous chromosomes are not perfectly colinear. Structural variations, differences in telomere and centromere lengths, and local polymorphisms can shift genomic coordinates even when corresponding genes are conserved [16, 37].

We analyzed 174 human DNA molecules and manually inspected their alignment results. Figure 6b summarizes the genome‐wide distribution of log(P) values obtained from aligning the 174 molecules across all 46 chromosomes. Green dots indicate alignments confirmed by manual review, whereas pink dots represent unvalidated or inconsistent alignments. All examined alignments with log(P) values below −9 were validated without exception. In contrast, when log(P) values were around −4, approximately half of the alignments were confirmed as correct. Although a complete reference genome specific to the HG02982 cells was not available—meaning that genuine structural differences from the hg002v1.1 reference may contribute to some discrepancies—50% of the molecules were confidently validated against the hg002v1.1 assembly.

Figure 6c compares the number of correctly top‐ranked molecules identified using cc_rg2, the composite scoring metric underlying DOM, and Maligner, which relies solely on barcode‐based single‐channel alignment. Among the 174 molecules analyzed, cc_rg2 correctly ranks 87 molecules (50%) at position 1, whereas Maligner correctly ranks 58 molecules (33%). Notably, only one molecule was correctly ranked first by Maligner but not by cc_rg2. The relative performance of Maligner compared to cc_rg2 corresponds to 66% (33%/50%), similar to the trend observed in the *E. coli* dataset (69% = 66%/95%) shown in Figure 4b. These results demonstrate that integrating complementary information channels improves genome‐wide single‐molecule alignment performance relative to barcode‐only methods.

**FIGURE 7:**
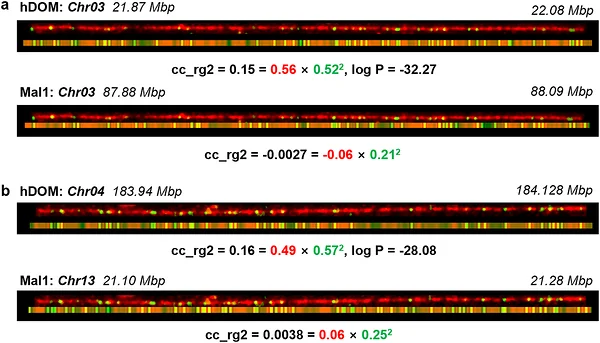
Accurate locus identification by hDOM compared to green barcode only alignment. (a) Example molecule correctly aligned by hDOM to chromosome 3 (21.87–22.08 Mbp) with a high combined score (cc_rg2 = 0.15 = 0.56 × 0.52^2^, log(P) = −32.27). In contrast, alignment using green‐label information alone (Mal1) incorrectly maps the same molecule to a distant region on chromosome 3 (87.88–88.09 Mbp), yielding a near‐zero combined score (cc_rg2 = −0.0027 = −0.06 × 0.21^2^). (b) A second example in which hDOM correctly assigns the molecule to chromosome 4 (183.94–184.13 Mbp) (cc_rg2 = 0.16 = 0.49 × 0.57^2^, log(P) = −28.08), whereas green‐channel–only alignment misidentifies the locus as chromosome 13 (21.10–21.28 Mbp) with a substantially lower score (cc_rg2 = 0.0038 = 0.06 × 0.25^2^).

Figure 7 shows representative examples in which hDOM and barcode‐only alignment (Maligner) produce different top‐ranked genomic loci. In both examples shown in Figure 7a,b, the two molecules were aligned by hDOM to chromosome 3 (log(P) = −32.27) and chromosome 4 (log(P) = −28.08), respectively, with highly distinctive placement scores. In contrast, alignment using barcode‐only mapping (Maligner) identified entirely different genomic loci as the top‐ranked candidates. Closer inspection of these cases reveals that in Figure 7a, the green‐channel correlation (cc_g = 0.21), and in Figure 7b, cc_g = 0.25, indicating that the green label patterns alone show moderate agreement with the incorrectly assigned loci. From a barcode‐only perspective, these matches appear plausible. However, when the red intensity correlation (cc_r) is considered, the loci identified by Maligner exhibit substantially lower concordance compared to those selected by hDOM. In contrast, the hDOM‐assigned positions display strong agreement in both green and red channels, resulting in substantially higher combined scores (cc_rg2) and lower log(P) values. These results demonstrate that reliance on discrete barcode similarity alone can lead to misalignment, particularly in a large genome such as the human genome. By integrating both label‐pattern information and AT intensity profiles, hDOM suppresses spurious matches and enhances locus specificity. Figure 7 therefore, illustrates how integration of barcode and AT‐profile information can improve locus discrimination relative to barcode‐only mapping approaches.

## 3 Conclusion

In this study, we developed DOM, a dual‐channel optical genome mapping approach that integrates AT‐frequency–dependent intensity profiling (cc_r) with sequence‐specific barcode labeling (cc_g) to enable robust and high‐confidence single‐molecule alignment. By combining sparse sequence anchors with dense backbone intensity information, DOM enhances locus discrimination beyond conventional barcode‐only mapping approaches. Importantly, the complementary nature of these two channels enables reliable alignment even in the presence of missing barcode labels or molecule‐to‐molecule heterogeneity in AT‐specific staining intensity, as information from one channel compensates for imperfections in the other.

Genome‐wide validation in both *E. coli* and the human genome demonstrates that cross‐correlation–derived placement scoring enables quantitative and scalable evaluation of alignment confidence. In diploid human genomes, DOM provides a quantitative framework for distinguishing candidate genomic loci arising from homologous redundancy and sequence similarity. By improving positional specificity without requiring deep molecular coverage, DOM strengthens the analytical power of optical genome mapping at the single‐molecule level.

While the present study focuses on establishing and validating the DOM framework, the increased information density provided by the dual‐channel approach may prove particularly valuable for structural variation detection and haplotype‐resolved mapping in large and repetitive genomes. With further advances in labeling efficiency, molecular throughput, and automated confidence‐based filtering, DOM may enable more comprehensive single‐molecule analysis of complex diploid genomes.

## 4 Experimental Section

### 4.1 Chemicals

DLE‐1 was obtained from Bionano Genomics (San Diego, CA, USA). The 5’‐6‐FAM labeled oligonucleotides used in FRET measurements were from Cosmogenetech (Seoul, Korea). Proteinase K and RNase I were obtained from Enzynomics (Daejeon, Korea), and β‐agarase I from New England Biolabs (Ipswich, MA, USA). ATTO647N NHS ester was purchased from ATTO‐TEC (Siegen, Germany). N‐trimethoxysilylpropyl‐N,N,N‐trimethylammonium chloride (50% methanol) was obtained from Alfa Aesar (Ward Hill, MA, USA). Ethanol (99.9%), sulfuric acid, and hydrogen peroxide were from Jin Chemical (Siheung, Korea), and N,N‐dimethylformamide (DMF) and N,N‐diisopropylethylamine (DIEA) were purchased from Sigma‐Aldrich.

### 4.2 Synthesis of ATTO647N‐β2‐Py4‐β‐Py4‐Dp (AP8)

ATTO647N‐β2‐Py4‐β‐Py4‐Dp (AP8) was synthesized as previously described [25]. Briefly, H_2_N‐β2‐Py4‐β‐Py4‐Dp (H_2_N‐P8) was prepared by solid‐phase synthesis. Subsequently, H_2_N‐P8 (1.3 mg, 1.0 µmol) and ATTO647N NHS ester (1.0 mg, 1.2 µmol) were dissolved in 200 µL of N,N‐dimethylformamide (DMF) and 0.50 µL of N,N‐diisopropylethylamine (DIEA, 2.4 µmol), followed by mixing at room temperature with protection from light. After the reaction was finished, the product was purified by high performance liquid chromatography (HPLC), followed by lyophilization of the collected fractions to afford AP8 as a blue powder.

### 4.3 Preparation of Positively Charged Glass Surface

The glass coverslips were prepared as previously described [38, 39]. Briefly, piranha‐cleaned glass surfaces were functionalized with N‐trimethoxysilyl propyl‐N,N,N‐trimethylammonium chloride to generate positively charged surfaces, which were stored in ethanol.

### 4.4 Preparation of E. coli Genomic DNA

*E. coli* K‐12 MG1655 cells were grown in LB medium at 37°C with agitation at 200 rpm until reaching OD_600_ = 0.6. The cells were pelleted by centrifugation at 10,000 × g for 10 min, washed twice with 1× PBS, and resuspended in the same buffer to OD_600_ = 2. The cell suspension was combined with molten 2% LMP agarose to produce a final agarose concentration of 0.7%, dispensed as 20 µL aliquots, and allowed to solidify at 4°C. The embedded cells were lysed with 2 mg/mL proteinase K at 42°C for 2 h, followed by a second treatment with freshly prepared enzyme. The gel plugs were subsequently washed several times with 1× TE buffer. To remove RNA, 200 U of RNase I was added and incubated for 1 h at 37°C.

### 4.5 E. coli Reference Genome Sequence

The genome sequence of *E. coli* strain used in this study was corrected by Illumina sequencing and Oxford Nanopore data. When compared to the NCBI *E. coli* str. K‐12 substr. MG1655 sequence (NC_000913.3), our lab strain exhibited 99.4% sequence identity (4,628,248/ 4,641,652 bp) with some variations. We employed the Canu program (https://github.com/marbl/canu) [40] to assemble the Illumina and Nanopore sequences, resulting in a final genome length of 4,629,724 bp. The complete sequence file (MG1655_Lab.fasta) is provided in the Supplementary Information (SI).

### 4.6 Preparation of HG02982 Genomic DNA

The HG02982 cell line, purchased from the Coriell Institute, was cultured in RPMI‐1640 medium supplemented with L‐glutamine (Corning), 15% fetal bovine serum (Gibco), and Antibiotic‐Antimycotic (Gibco). The cells were harvested by centrifugation at 300 g for 10 min, washed twice with PBS, and resuspended in the same buffer. The cell suspension was mixed with molten 2% LMP agarose solution to achieve a final concentration of 0.7%. This mixture was dispensed as 20 µL droplets and solidified at 4°C. Cells within a gel plug were lysed by 2 mg/mL proteinase K for 2 h at 42°C. The proteinase K reaction was repeated with freshly prepared enzyme. The gel plugs were washed several times with 1×TE. To remove RNA from the gel plugs, 200 U of RNase I was added, and incubated for 1 h at 37°C. Two gel plugs (40 µL) were used for the subsequent experiment.

### 4.7 Labeling and Staining of Genomic DNA

Genomic DNA extracted from either *E. coli* or HG02982 was labeled using the Bionano prep direct label and stain (DLS) protocol with minor modifications. Initially, DNA within an agarose plug was labeled by DLE‐1 and DL‐green dye. After the labeling reaction, the sample was treated with proteinase K to digest residual enzymes, and then the gel plugs were thoroughly washed using 1×TE buffer to remove residual DL‐green dyes. The agarose plugs containing DNA were melted at 65°C for 15 min. The molten agarose was cooled to 42°C and incubated with 1 U of β‐agarase I (NEB) for 1 h. β‐agarase I was heat‐inactivated at 65°C for 15 min. The DNA solution was mixed with 1.7 µM AP8 in a 1:1 volume ratio at RT for 30 min. Before observation, this mixture was diluted tenfold using 1×TE.

### 4.8 Microscopy

An inverted microscope (Olympus IX70) equipped with a 100× UPlanSApo oil immersion objective was used for single‐molecule observations. Illumination was provided by an LED light source (SOLA SM II light engine, Lumencor, Beaverton, OR, USA). Fluorescence images were captured using a scientific complementary metal‐oxide‐semiconductor camera (Prime sCMOS, Teledyne Photometrics, AZ, USA) and saved in a 16‐bit TIFF format using the Micro‐manager software. Image processing was performed using ImageJ and Python programs developed in our lab.

### 4.9 Affinity Measurement of AP8 to the AT Repeated Sequence Using FRET

The binding affinities between AP8 and DNA oligonucleotides containing 6‐FAM (fluorescein amidite) at the 5′‐terminal were determined through FRET analysis [32, 41]. Each oligonucleotide contained AT base pairs ranging from 0 to 10‐mer, and their sequences are listed in Table S1. For this experiment, each oligonucleotide was diluted to 400 nM using 1×TE buffer and then mixed with AP8 at concentrations of 1.6 µM in a 1:1 volume ratio. After incubating the mixtures at room temperature for 30 min, the fluorescence intensities were measured through a Hitachi F‐7000 fluorometer (Hitachi, Japan).

### 4.10 Alignment Python Programs

All Python codes are available in the GitHub repository (https://github.com/jolaboffice/DOM). The sim2RGmap.py produces the red‐channel map via AP8 binding simulations and the green‐channel map using Bio.Seq.find(). For a simpler and faster implementation, seq2RGmap.py generates a two‐channel in silico map from a FASTA file based on local A/T frequency. DOM.py serves as the main script that performs genome‐wide alignment and visualization for dual‐channel DOM images. For each DNA molecule, the program computes the red‐channel correlation and green‐channel correlation across multiple stretch factors and genomic positions, and identifies candidate alignment sites based on the combined score. The program employs PCC for continuous AT‐intensity profiles and DCC for discrete sequence‐specific labels, as DCC more effectively captures the positional correspondence of sparse green‐label signals [21]. PCC evaluates similarity in overall signal shape after mean normalization, whereas DCC directly measures the positional overlap between discrete labeling patterns. The two correlation scores are defined as:

$$pcc\left(x,y\right)=\frac{\sum \left(x_{i}-\bar{x}\right)\left(y_{i}-\bar{y}\right)}{\sqrt{\sum {\left(x_{i}-\bar{x}\right)}^{2}}\sqrt{\sum {\left(y_{i}-\bar{y}\right)}^{2}}}$$

$$dcc\left(x,y\right)=\frac{\sum x_{i}y_{i}}{\sqrt{\sum x_{i}^{2}}\sqrt{\sum y_{i}^{2}}}$$

Candidate loci were ranked using the combined score cc_rg2 = cc_r × cc_g^2^. For each molecule, the genome‐wide distribution of cc_rg2 values obtained from all scanned genomic positions was characterized by its mean (m) and standard deviation (σ). A standardized score was calculated as:

$$Z=\frac{X-m}{\sigma }$$

where X denotes the observed cc_rg2 value for a candidate alignment. A genome‐wide placement score (P) was then calculated as:

$$P=1-\Phi \left(Z\right)$$

where Φ denotes the cumulative distribution function of the standard normal distribution. Lower placement scores indicate greater separation from the genome‐wide background distribution and therefore higher alignment distinctiveness. Because the underlying cc_rg2 distribution does not strictly follow a normal distribution, P is used here as a placement score describing alignment distinctiveness rather than as a formal parametric *p*‐value.

The @numba.njit decorator was applied to accelerate the PCC and DCC computations by compiling the Python code for numerically intensive operations. Maligner [36], a dynamic programming‐based aligner downloaded from GitHub (https://github.com/LeeMendelowitz/maligner), was used for comparative analysis of barcode‐based alignment results. A Python 3–compatible version of Maligner has been generated and included in our GitHub repository (https://github.com/jolaboffice/DOM).

### 4.11 In Silico Generation of Ap8 Fluorescence Profiles

The sim2RGmap.py script generates simulated AP8 fluorescence profiles from DNA sequences. Binding events were modeled by allowing AP8 to associate probabilistically with DNA according to a sequence‐dependent affinity model derived from experimentally measured AP8 binding preferences (Figure 1), generating a spatial distribution of fluorescent signals. A bound AP8 molecule was assumed to occupy a finite DNA segment (binding length), and neighboring binding events within an exclusion window (block length) were suppressed to account for steric hindrance. In the simulation, candidate binding sites were randomly shuffled and sequentially filtered using the block‐length exclusion window, thereby enforcing steric exclusion without repeated rejection sampling. The PSF was modeled as a Gaussian function to emulate the optical characteristics of the microscope. Final simulations were performed using the AP8 affinity table derived from the independent FRET measurements (Figure 1). The @numba.njit decorator was applied to accelerate the numerical computations in sim2RGmap.py.

## Author Contributions

**Kaori Hashiya**: methodology. **Jinhui Hong**: methodology. **Kyubong Jo**: project administration, funding acquisition, investigation, conceptualization, supervision, writing – review and editing. **Jaeyoung Bae**: investigation, methodology, formal analysis, validation, visualization, writing – original draft, writing – review and editing, data curation. **Yunchul Kim**: software, formal analysis, data curation, investigation, writing – review and editing, validation, methodology, visualization. **Hiroshi Sugiyama**: methodology. **Toshikazu Bando**: methodology. **Joohee Choe**: software, visualization, methodology, formal analysis, data curation, investigation, writing – review and editing, validation. **Jung Heon Lee**: supervision, funding acquisition, project administration, writing – review and editing. **Seonghyun Lee**: formal analysis, writing – original draft, writing – review and editing, project administration. **Byoungsang Lee**: software.

## Funding

Samsung Research Funding Center (SRFC‐MA2101‐07), and National Research Foundation of Korea (NRF) (RS‐2024‐00441954, and RS‐2025‐00515166).

## Conflicts of Interest

The authors declare no conflicts of interest.

## Supporting information




**Supporting File**: smtd70883‐sup‐0001‐SuppMat.docx.

## Data Availability

The data that support the findings of this study are available on request from the corresponding author. The data are not publicly available due to privacy or ethical restrictions.
