# Supplementary material for: DOM: Dual Optical Mapping Integrating Sequence‐Specific Barcodes and A/T Density Profiles
Source: Small Methods. 2026 Jul 22;10(16):e70883. doi: 10.1002/smtd.70883 (PMC13505701; doi:10.1002/smtd.70883)
Supplement: Supplementary file 1 [file SMTD-10-e70883-s001.docx]

Supporting Information

DOM: Dual Optical Mapping Integrating Sequence-Specific Barcodes and A/T Density Profiles

Jaeyoung Bae^1†^, Yunchul Kim^2†^, Joohee Choe^1†^, Jinhui Hong^1^, Byoungsang Lee^2^, Kaori Hashiya^3^, Toshikazu Bando^3^, Hiroshi Sugiyama^3^, Seonghyun Lee*^4,5,6^, Jung Heon Lee*^2,4^, Kyubong Jo*^1,7^

^1^ Department of Chemistry, Sogang University, Seoul, Korea

^2^ School of Advanced Materials Science and Engineering, Sungkyunkwan University, Suwon, Korea
^3^ Institute for Integrated Cell-Material Sciences (iCeMS), Kyoto University, Kyoto, Japan

^4^ Department of MetaBioHealth, Sungkyunkwan University, Suwon, Korea

^5^ Department of Precision Medicine, School of Medicine, Sungkyunkwan University, Suwon, Korea

^6^ Department of Chemical and Biomolecular Engineering, Sogang University, Seoul, Korea

^7^ Center for Nano Materials, Sogang University, Seoul, Korea

* To whom correspondence should be addressed. Tel: +82-2-705-8881;
Email: [jokyubong@sogang.ac.kr](mailto:jokyubong@sogang.ac.kr);

Correspondence may also be addressed to: [jhlee7@skku.edu](mailto:jhlee7@skku.edu); shlee9@skku.edu

**Supplementary Table and Figures**

**Table S1. Sequence of oligonucleotides used in FRET experiment**

**Figure S1. Comparison of cc_r values using three reference maps (SIM, W1, and W6).**

**Figure S2. List of genes between the gaps in Figure 1a**

**Figure S3. Distortion-induced misalignment and correction by cropping**

**Figure S4. cc_rg2 and placement scores corresponding to the data plotted in Figure 4d.**

**Supplementary Table and Figures**

| **Name** | **Oligonucleotide Sequence** |
| --- | --- |
| 0AT | 5’-6-FAM-GGC CGC GCG CGC GCC CGG TTT TCC GGG CGC GCG CGC GGC C |
| 1AT | 5’-6-FAM-GGC CGC GC**A** GCG CGC CGG TTT TCC GGC GCG C**T**G CGC GGC C |
| 2AT | 5’-6-FAM-GGC CGC GC**A T**CG CGC CGG TTT TCC GGC GCG **AT**G CGC GGC C |
| 3AT | 5’-6-FAM-GGC CGC G**AT A**CG CGC CGG TTT TCC GGC GCG **TAT** CGC GGC C |
| 4AT | 5’-6-FAM-GGC CGC G**AT AT**G CGC CGG TTT TCC GGC GC**A TAT** CGC GGC C |
| 5AT | 5’-6-FAM-GGC CGC G**AT ATA** CGC CGG TTT TCC GGC G**TA TAT** CGC GGC C |
| 6AT | 5’-6-FAM-GGC CGC G**AT ATA T**GC CGG TTT TCC GGC **ATA TAT** CGC GGC C |
| 7AT | 5’-6-FAM-GGC CGC G**AT ATA TA**C CGG TTT TCC GG**T ATA TAT** CGC GGC C |
| 8AT | 5’-6-FAM-GGC CGC G**AT ATA TAT** CCG GTT TTC CGG **ATA TAT AT**C GCG GCC |
| 9AT | 5’-6-FAM-GGC CGC G**AT ATA TAT A**CC GGT TTT CCG G**TA TAT ATA T**CG CGG CC |
| 10AT | 5’-6-FAM-GGC CGC G**AT ATA TAT AT**C CGG TTT TCC GG**A TAT ATA TAT** CGC GGC C |

Table S1. Sequence of oligonucleotides used in FRET experiment

**Figure S1.** Comparison of cc_r values using three reference maps (SIM, W1, and W6). (a) cc_r values calculated from full-length regions of 172 *E. coli* DNA clips that were ranked first by cc_rg2. The SIM reference map consistently yields higher cc_r values than W1 or W6. (b) cc_r values calculated from central 50 kb of the same 172 *E. coli* DNA clips that were ranked first by cc_rg2. (c) Python program used to calculate cc_r for three reference maps. The grey-shaded region indicates the central 50 kb used for the analysis in (b). (d) Representative subset of the raw cc_r data corresponding to (a) and (b), illustrating that the SIM reference map consistently provides the highest cc_r values for most molecules.

**Figure S2.** List of genes between the gaps in Figure 3a. The smallest gap (3.1 kb) contains five genes and the largest gap (93.2 kb) contains 91 genes. Detailed sequence information might be missing when solely using barcode-based optical mapping. Reference genome: NC_000913.3.

**Figure S3.** Distortion-induced misalignment and correction by cropping. Each example shows a DNA molecule with local folding, aggregation, or partially unstretched regions that suppress cc_r and cc_g, resulting in low initial cc_rg2 ranks. The distorted segment is marked with cyan boxes. For each molecule, the yellow arrow reports the original cc_rg2 value and rank obtained using the full-length image, whereas the green arrow shows the improved cc_rg2 value and updated rank after cropping out the distorted region.

**Figure S4.** cc_rg2 and placement scores corresponding to the data plotted in Figure 4d.
